# Do genetic predictors of pain sensitivity associate with persistent widespread pain?

**DOI:** 10.1186/1744-8069-5-56

**Published:** 2009-09-23

**Authors:** Kate L Holliday, Barbara I Nicholl, Gary J Macfarlane, Wendy Thomson, Kelly A Davies, John McBeth

**Affiliations:** 1 Arthritis Research Campaign Epidemiology Unit, University of Manchester, Manchester, UK; 2 Aberdeen Pain Research Collaboration (Epidemiology Group), School of Medicine and Dentistry, University of Aberdeen, Aberdeen, UK

## Abstract

Genetic risk factors for pain sensitivity may also play a role in susceptibility to chronic pain disorders, in which subjects have low pain thresholds. The aim of this study was to determine if proposed functional single nucleotide polymorphisms (SNPs) in the *GTP cyclohydrolase *(*GCH1*) and *μ opioid receptor *(*OPRM1*) genes previously associated with pain sensitivity affect susceptibility to chronic widespread pain (CWP). Pain data was collected using body manikins via questionnaire at three time-points over a four year period from subjects aged 25-65 in the North-West of England as part of a population based cohort study, EPIFUND. CWP was defined at each time point using standard criteria. Three SNPs forming a proposed "pain-protective" haplotype in *GCH1 *(rs10483639, rs3783641 and rs8007267) and two SNPs in *OPRM1 *(rs1777971 (A118G) and rs563649) were genotyped in cases with persistent CWP (CWP present at ≥2 time-points) and controls who were pain-free at all time-points. The expectation-maximisation algorithm was used to estimate haplotype frequencies. The frequency of the "pain-protective" (CAT - C allele of rs10483639, A allele of rs3783641 and T allele of rs8007267) haplotype was compared to the frequency of the other haplotypes between cases and controls using the χ^2 ^test. Allele frequencies and carriage of the minor allele was compared between cases and controls using χ^2 ^tests for the *OPRM1 *SNPs. The frequency of the proposed *GCH1 *"pain-protective" haplotype (CAT) did not significantly differ between cases and controls and no significant associations were observed between the *OPRM1 *SNPs and CWP. In conclusion, there was no evidence of association between proposed functional SNPs, previously reported to influence pain sensitivity, in *GCH1 *and *OPRM1 *with CWP. Further evidence of null association in large independent cohorts is required to truly exclude these SNPs as genetic risk factors for CWP.

## Findings

The full aetiology of chronic widespread pain (CWP) is unknown but evidence suggests that genetic risk factors may contribute [1]. As a complex trait it is likely that multiple genes will be involved with each of them having a small effect on the phenotype. Sensitivity to painful stimuli has also been shown to be genetically influenced [2,3]. The relationship between CWP and pain sensitivity is unclear. Clinic studies have shown increased pain sensitivity in fibromyalgia patients using measures such as thermal and mechanical stimuli and temporal summation [4-8]. However, it is unknown whether the relationship between CWP and pain sensitivity is causal or consequential. Genes which influence pain sensitivity may also be important in susceptibility to chronic pain syndromes such as CWP. Previous studies have reported associations between known and purported functional polymorphisms and pain sensitivity for two genes, *GCH1 *[9-11] and *OPRM1*[12,13].

*GCH1 *encodes GTP cyclohydrolase which is a pathway synthesis enzyme for Tetrahydrobiopterin (BH4), a cofactor essential in neurotransmitter synthesis which is up-regulated in neuropathic and inflammatory pain [9]. In 2006, a haplotype comprised of 15 SNPs spanning *GCH1 *was reported to associate with a reduced hyperalgesic response to a mechanical pain stimulus and also with lower levels of lumbar pain following diskectomy [9], thus implicating *GCH1 *as a potentially important gene in both pain sensitivity and chronic pain. In 2007, Lotsch et al demonstrated that this haplotype could be captured with 100% specificity and sensitivity by genotyping only three single nucleotide polymorphisms (SNPs); rs10483639, rs3783641 and rs8007267, with the C, A and T alleles of these three SNPs respectively forming the "pain-protective" haplotype. Subsequent studies, however, have yielded conflicting results with some confirming the protective effect of the haplotype on pain sensitivity [10,11] and others not [14]. Null associations between the haplotype and pain severity following dental surgery [14] and in subjects with pancreatitis [15] have also been reported. Tegeder et al (2006) also reported lower levels of *GCH1 *mRNA in subjects carrying the "pain-protective" haplotype [9]. A subsequent study also found that subjects homozygous for the CAT haplotype had reduced levels of *GCH1 *mRNA as well as reduced levels of BH4 compared to subjects not carrying the haplotype [10]. In addition, Zhang et al (2007) found that the 3' UTR SNP rs841, which is in LD with the "pain-protective" haplotype, also affects expression of the gene [16].

The second gene of interest, *OPRM1*, encodes the μ opioid receptor which binds both exogenous and endogenous opiates. The variant G allele of the non-synonymous SNP, A118G (rs1799971) which changes asparagine to aspartic acid, increases the ability of the receptor to bind β-endorphin and has been associated with increased pain thresholds [12]. More recently, Shabalina et al (2009) conducted a comprehensive analysis of the variation within *OPRM1 *and although they did not observe an association between A118G and pain sensitivity they found the minor (T) allele of rs563649 to be associated with increased pain sensitivity. They also found that rs563649 is located within the 5'UTR of an isoform of the μ opioid receptor in a putative internal ribosome entry site (IRES). In vitro, the T allele increased translational activity through increased ribosome binding and lowered mRNA levels suggesting the SNP may be functionally important [13].

The aim of this study was to determine if the *GCH1 *"pain-protective" haplotype and the *OPRM1 *SNPs, rs563649 and rs1799971 are associated with CWP in a UK population-based cohort.

Subjects, aged 25-65 were recruited from 3 primary care registers in the North-west of England into a prospective population-based cohort study (EPIFUND, Epidemiology of Functional Disorders). Pain data was collected at 3 time-points over a 4 year period via a postal survey. A detailed pain questionnaire and body manikins were used to ascertain CWP status using American College of Rheumatology Criteria (pain for ≥3 months in contra-lateral body quadrants above and below the waist and in the axial skeleton) at each time-point. DNA was obtained, using buccal swab sampling, from 1189 subjects who participated at all three time-points and had complete pain data. From this study population, a nested case-control study was conducted with cases being subjects who had CWP for ≥2 time-points and controls were subjects who were pain-free at all 3 time-points. There was a female preponderance (58%) in subjects included in the analysis and the mean ± standard deviation age of subjects was 50 ± 9.6 years old. The proportion of females did not significantly differ between cases and controls (p > 0.05) and cases were significantly older than controls (p < 0.01). Ethnicity was not determined in EPIFUND; however, subjects were recruited from a predominantly white Caucasian geographic area.

The three SNPs in *GCH1*; rs10483639, rs3783641 and rs8007267, which capture the pain protective haplotype [17] and two SNPs in *OPRM1*; rs563649 and rs1799971 (A118G), were genotyped in all cases (n = 197) and all controls (n = 197). Genotyping was carried out using Sequenom MassArray technology. Pair-wise LD was examined using Haploview [18]. All SNPs were in Hardy-Weinberg Equilibrium and their genotyping success rate was ≥97%.

**Figure 1:**
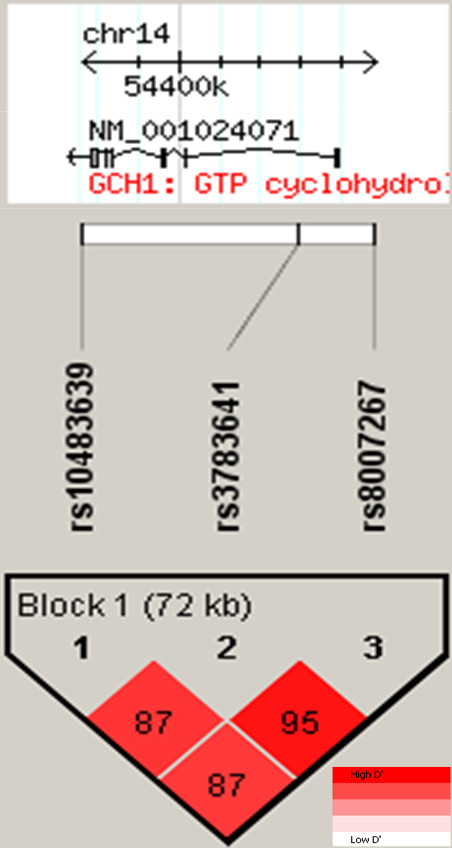
**Linkage disequilibrium of the *GCH1 *haplotype**. SNPs genotyped and their position in *GCH1 *is shown with pair-wise LD (coloured by D' and numbered with r^2 ^values).

The genomic position of the three *GCH1 *SNPs and the pair-wise LD between them in this UK population is shown in Figure 1. Haplotype frequencies in the cases (N = 164) and controls (N = 172) were estimated using the Expectation-Maximisation algorithm and compared using a χ^2^test in PLINK [19]. Allele frequencies were compared between cases and controls for the *OPRM1 *SNPs using a χ^2 ^test. The effect of having one or two copies of the minor allele compared to zero copies (dominant model) was also tested using a χ^2 ^test. Odds ratios (OR) and 95% confidence intervals (95%CI) were calculated using logistic regression. Analysis was conducted using STATA version 9.2.

Two common, GTC (76%) & CAT (17%); and two rare (≤3%), CTC and CAC; *GCH1 *haplotypes were identified in the population. There was no significant difference in the overall distribution of haplotypes between cases and controls (p = 0.488). The pain-protective (CAT) haplotype was less frequent in cases (15%) compared to controls (19%), although this was also non-significant (p = 0.129). Concurrently the most common haplotype, GTC, showed a non-significant (p = 0.190) increased frequency in cases (81%) compared to controls (77%) (Table 1). This result is in keeping with the findings of previous reports [14,15] that did not replicate the findings of the initial study which observed association between the haplotype and both pain sensitivity and chronic pain [9].

**Table 1:** Results of haplotype analysis of *GCH1 *with CWP

**Combination of SNPs**	**Overall distribution** **p-value**	**Haplotype**	**Frequency of individual haplotypes**		
			
			**Cases**	**Controls**	**p-value**
**rs10483639 - rs3783641 - rs8007267**	0.488	**GTC**	0.81	0.77	0.190
		**CAT**	0.15	0.19	0.129
		**CTC**	0.03	0.02	0.714
		**CAC**	0.02	0.02	0.879

There was no evidence of association with CWP for either of the *OPRM1 *SNPs, although rs563649 showed a trend towards a protective effect of the T allele (Table 2). This is in contrast to the findings of Shabalina et al (2009) who found the T allele to be associated with increased pain scores [13].

**Table 2:** Results of analysis of *OPRM1 *SNPs with CWP

**SNP**			**Cases**	**Controls**	**OR (95%CI)**	**p-value**
			**N (%)**	**N (%)**		
**rs563649**	**Allele**	**C**	336 (92)	321 (90)	1	
		**T**	30 (8)	37 (10)	0.77 (0.45, 1.32)	0.321
						
	**Genotype**	**CC**	155 (85)	146 (82)	1	
		**CT & TT**	28 (15)	33 (18)	0.80 (0.46, 1.39)	0.425
						
**rs1799971**	**Allele**	**A**	287 (90)	298 (90)	1	
		**G**	31 (10)	34 (10)	0.95 (0.55, 1.63)	0.834
						
	**Genotype**	**AA**	130 (82)	136 (82)	1	
		**AG & GG**	29 (18)	30 (18)	1.01 (0.58, 1.78)	0.967

The aim of this study was to determine if putative functional SNPs reported to influence pain sensitivity are genetic predictors of CWP susceptibility. There was no evidence of a significant association between the *GCH1 *"pain-protective" haplotype or the two SNPs in *OPRM1 *and CWP. It should be noted that sample size in the studies of pain sensitivity and chronic pain conducted on these two genes were relatively small with the maximum being N = 632. Equally in this study the sample size was modest therefore the decreased prevalence of the *GCH1 *CAT haplotype in subjects with persistent CWP compared to pain-free controls may not have reached significance in this study due to limited power. Further investigation of this relationship in larger independent cohorts is therefore warranted.

## Competing interests

The authors declare that they have no competing interests.

## Authors' contributions

KL & BN were both involved in study design, DNA extraction and genotyping, statistical analysis and drafting of the manuscript. WT contributed to the design of the study and interpretation of data. JM contributed to conception and design of the study, acquisition and interpretation of the data. GM contributed to the conception of the study and acquisition of data. KD contributed to conception and design of the study. All authors contributed to the revision of the manuscript and approved the final version.
